# Nitrogen-fixing bacteria promote growth and bioactive components accumulation of *Astragalus mongholicus* by regulating plant metabolism and rhizosphere microbiota

**DOI:** 10.1186/s12866-024-03409-y

**Published:** 2024-07-15

**Authors:** Shi Zhiyong, Guo Yaxuan, Wang Yuanyuan, Yan Xiang, Guo Xu, Lei Zhenhong, Niu Jingping, Liang Jianping, Li Zhenyu

**Affiliations:** 1 College Of Life Sciences, Shanxi Agricultural University, Jinzhong, 030801 China; 2 Modern Research Center for Traditional Chinese Medicine, Shanxi University, Taiyuan, 030006 China; 3 Shanxi Key Laboratory of Chinese Veterinary Medicine Modernization, Shanxi Agricultural University, Jinzhong, 030801 China; 4 Shanxi Zhendong Pharmaceutical (China), Changzhi, 047000 China

**Keywords:** *Astragalus mongholicus*, PGPR, Biological nitrogen fixation, Medicinally active components, Non-targeted metabolomics, 16S rRNA sequencing, Bacterial community

## Abstract

**Background:**

The excessive application of chemical fertilizers in the cultivation of *Astragalus mongholicus Bunge* results in a reduction in the quality of the medicinal plant and compromises the sustainable productivity of the soil. PGPB inoculant is a hot topic in ecological agriculture research. In the cultivation of *Astragalus mongholicus*, the screened nitrogen-fixing bacteria can promote plant growth, however, whether it can promote the accumulation of main bioactive components remains unknown. In this study, mixed inoculants containing 5 strains of growth promoting bacteria (*Rhizobium T16* *,* *Sinorhizobium T21* *,* *Bacillus J1* *,* *Bacillus G4 and Arthrobacter J2*) were used in the field experiment. The metabolic substances in the root tissues of *Astragalus mongholicus* were identified during the harvest period by non-targeted metabolomics method, and the differential metabolites between groups were identified by statistical analysis. Meanwhile, high-throughput sequencing was performed to analyze the changes of rhizosphere soil and endophytic microbial community structure after mixed microbial treatment.

**Results:**

The results of non-targeted metabolism indicated a significant increase in the levels of 26 metabolites after treatment including 13 flavonoids, 3 saponins and 10 other components. The contents of three plant hormones (abscisic acid, salicylic acid and spermidine) also increased after treatment, which presumed to play an important role in regulating plant growth and metabolism. Studies on endosphere and rhizosphere bacterial communities showed that Rhzobiaceae, Micromonosporaceae, and Hypomicrobiaceae in endophytic, and Oxalobactereae in rhizosphere were significantly increased after treatment. These findings suggest their potential importance in plant growth promotion and secondary metabolism regulation.

**Conclusions:**

This finding provides a basis for developing nitrogen-fixing bacteria fertilizer and improving the ecological planting efficiency of *Astragalus mongholicus.*

**Supplementary Information:**

The online version contains supplementary material available at 10.1186/s12866-024-03409-y.

## Background

*Astragalus mongholicus (Huangqi),* a valuable traditional Chinese medicinal herb, is primarily utilized for its root, which possesses significant medicinal properties. It is also a popular food for specified health uses. The key bioactive constituents found in *A. mongholicus* encompass flavonoids, saponins, polysaccharides, amino acids, and diverse trace elements [1–3]. In order to increase the yield of *A. mongholicus* and bring higher economic benefits, the traditional agricultural planting methods, which rely on chemical fertilizers and pesticides, have been used. These practices are not only costly, but also lead to soil acidification, a decline in organic matter content, and a reduction of rhizosphere microbial diversity, making large-scale cultivation unsustainable [4].

Soil microorganisms play a crucial role in nutrient cycling, maintaining system stability, enhancing resistance to interference, and promoting sustainable productivity within the soil ecosystem [5]. Extensive research has demonstrated the positive effects of various microbial fertilizers on major crops, effectively improving nutrient utilization efficiency and increasing plant yield in an economically viable, efficient, and environmentally friendly manner [6–8]. Plant-growth-promoting bacteria (PGPB) are of particular significance in supporting plant growth and health, and their utilization as microbial fertilizers represents a promising alternative for sustainable and environmentally conscious agricultural practices [9].

The utilization rate of nitrogen fertilizer in plants is low, and most of the amended nitrogen pollutes the atmosphere and deteriorates water quality due to ammonia volatilization, denitrification and nitrate leaching [10]. Biological nitrogen fixation is a vital process that contributes to the availability of nitrogen in soil. It involves the utilization of symbiotic and non-symbiotic nitrogen-fixing microorganisms, which provide plants with a crucial source of nitrogen. *A. mongholicus*, being a perennial leguminous plant, forms nodules through symbiosis with rhizobium, thus facilitating enhanced nutrient absorption in the plant [11]. Consequently, investigating the biological nitrogen fixation of *A. mongholicus* and its impact on yield and quality holds significant importance.

Microbial agents mainly refer to the manual screening or cultivation of microorganisms with specific functions, which can be made into living bacteria with high activity after processing, for the purpose of regulating plant rhizosphere environment and degrading harmful substances [12]. The research and application of host specific and efficient compound microbial nitrogen fixing bacteria is the focus of current biological nitrogen fixation research [13]. It is mainly used by a variety of screened host specific and efficient nitrogen fixing bacteria or by combining nitrogen fixing bacteria with plant-growth-promoting bacteria (PGPB), which not only stabilize the effect of nitrogen fixation, but also offers additional benefits to plants [14, 15].

Metabolomics is a powerful tool with a wide range of applications in studying and analyzing plant secondary metabolites [5, 16]. It provides a holistic view of the metabolic profile of plants, allowing for the identification and quantification of a broad range of metabolites. Plant secondary metabolites, as effective components, are important mediators of plant microbial interactions. The host related microbial community structure is regulated by some secondary metabolites, and the change of microbial community would also influence the synthesis of secondary metabolites [17–20]. The interaction between the microorganism and the host is presumed to promote the accumulation of bioactive components in medicinal plants [21, 22].

In previous experiments, strains of rhizobia from the root nodules of *A. mongholicus* were isolated. These strains, specifically *Rhizobium sp. T16* and *Sinorhizobium sp. T21*, demonstrated significant nitrogen fixation activity [23, 24]. Plant-growth-promoting bacteria *Bacillus sp. J1*, *Arthrobacter sp. J2* and *Bacillus sp. G4* with nitrogen fixation were isolated from root tissue and rhizosphere soil. The results showed that these two rhizobia and three PGPB strains could significantly promote the growth of *A. mongholicus* [23]. However, the effects of five strains as combined bacteria inoculants on the accumulation of main active components of *A. mongholicus* and the rhizosphere and root endophytic bacterial community are not clear.

This study objectives to: (1) study the effects of inoculation of synthetic bacteria agent (*Rhizobium*, *Bacillus* and *Agrobacterium*) on the growth and accumulation of secondary metabolites in *A. mongholicus*; (2) evaluate the effect of inoculation of synthetic bacteria on rhizosphere and root endophytic microbial communities; and (3) explore the relationship between the change of microbial community structure caused by inoculation of synthetic bacteria and the effective components accumulation of *A. mongholicus*. We hypothesized that synthetic nitrogen fixing bacteria agents may affect the root endophytic and rhizosphere microbiota, thereby comprehensively affecting the secondary metabolism of *A. mongholicus*.

## Materials and methods

### Bacterial strains and their culture

Two strains of host specific rhizobia, namely T16 (*Rhizobium sp. T16*) and T21 (*Sinorhizobium sp*. *T21*) were isolated from the root nodule of *A. mongholicus* and three high efficient nitrogen fixing bacteria, namely J1 (*Bacillus sp*. *J1*), J2 (*Arthrobacter sp*. *J2*) and G4 (*Bacillus sp. G4*) were isolated from the root tissue and rhizosphere soil of *A. mongholicus* in Hunyuan County, Shanxi Province, China. These bacteria were inoculated into Yeast Mannitol Broth (YMB) liquid medium for fermentation at 28 ℃ 48 h under static conditions for further experiments.

### Antagonistic test of strains

Two pairs of strains to be tested were inoculated on YMB solid plate medium vertically and horizontally, then cultured at 28 ℃ for 24 h. The bacterial growth was monitored at the intersection of the vertical line and the horizontal line. The inhibition of growth at the intersection suggests an antagonistic effect between the strains, rendering them unsuitable for mixed culture. Conversely, the absence of growth inhibition at the intersection indicates a lack of antagonistic effect, making them suitable for mixed culture.

### Preparation of bacterial inocula and inoculation process

Five strains of high efficiency nitrogen fixing bacteria (J1, J2, G4, T16, and T21) of *A. mongholicus* were cultured in sterilized YMB liquid culture medium with 250 mL conical flasks respectively on a shaker at 28℃ and 180 r/min for 48 h, and then made into bacterial stock solution for standby. The concentration of bacterial solution was approximately 1 × 10^10^ CFU.mL^−1^. The bacterial stock solution components were mixed in proportion and diluted with distilled water to form liquid bacterial agent (bacterial content was approximately 10^8^ CFU.mL^−1^). The experimental site was in Hunyuan County, Shanxi Province (39^◦^516 N, 113^◦^643 E, altitude: 1572 m). The new *A. mongholicus* seed from Hunyuan County was used as the test material. The seeds were first soaked in bacterial fertilizer solution for one hour to form inoculation, and then sowed in the experimental field (distilled water as control treatment). The area of each experimental plot was 100 m^2^ and the sowing amount was 500 g with a 30 cm row spacing. The experiment was repeated thrice. Random sampling of rhizosphere soil and root tissue were performed at 30, 60, and 120 days after seeding, corresponding to seedling stage, rapid growth stage and defoliation stage of *A. mongholicus* respectively.

### Illumina miseq sequencing and data processing of 16S rRNA

Total DNA of root tissue and rhizosphere soil samples were extracted using an Omega E.Z.N.A DNA extraction kit. The bacterial 16S rRNA genes were amplified using the universal primer pair F338/R806 (F338, 5′-ACTCCTACGGGAGGCAGCAG-3′; R806, 5′-GGACTACHVGGGTWTCTAAT-3′) for rhizosphere soil samples and F799/R1193 (F799, 5′-AACMGGATTAGATACCCKG-3′; R1193, 5′-ACGTCATCCCCACCTTCC-3′) for root tissue samples. PCR reactions were conducted using a 20 µL reaction system containing TransStart Fastpfu DNA Polymerase, following the specified conditions: an initial denaturation step at 95 ℃ for 2 min, followed by 35 amplification cycles consisting of denaturation at 95 ℃ for 30 s, annealing at 55 ℃ for 1 min, extension at 72 ℃ for 1 min, and a final extension step at 72 ℃ for 10 min. The resulting PCR products were purified and subjected to paired-end sequencing on an Illumina MiSeq PE300 platform (Illumina, Inc., Santiago, CA, USA) at Majorbio Bio-pharm Technology Co., Ltd (Shanghai, China).

The Illumina raw reads obtained were processed using QIIME2 microbial analysis platform [25]. Initially, the paired-end reads were merged and subsequently renamed based on the sample barcode. Clean reads were obtained after removing chimeric sequences. These clean reads were then clustered into operational taxonomic units (OTUs) using a 97% similarity threshold. To assess the similarities between bacterial communities, non-metric multidimensional scaling (NMDS) analysis was performed. The taxonomic information of species at each level was obtained through annotation results. LEfSe was employed to identify functional categories that were significantly enriched in different treatment groups [26]. A *p* value < 0.05 and a linear discriminant analysis (LDA) score > 2 were used as criteria for significance. Differential bacteria were identified between groups using a t-test (*p* < 0.05) and fold change (FC > 1.2 or < 0.8). Functional profiling of bacterial community was predicted using PICRUSt2 [27] and Greengenes database [28].

### Untargeted metabolome assay of root tissue

#### Materials and reagents

Ultrapure water was generated using a Milli-Q water purification system from Millipore (USA). LC–MS grade acetonitrile and formic acid were purchased from Thermo Fisher Scientific (Fairlawn, NJ, USA). Analytical grade methanol was procured from Tianjin Damao Chemical Reagent Factory.

#### Preparation of sample solutions

3 mL 50% aqueous methanol (v/v) was added to the fine powder of AR (0.15 g). The mixture was subjected to ultrasonication at room temperature for 20 min. Subsequently, the extracts were centrifuged at 3500 rpm for 10 min. The supernatant obtained after centrifugation was filtered through a 0.22 µm microporous membrane for subsequent analysis using ultra-high-performance liquid chromatography (UHPLC) and quadrupole time of flight mass spectrometry (Q-TOF–MS). Furthermore, for quality control (QC) purposes, 1 mL of each sample was combined to create a QC sample.

#### UHPLC/Q-TOF–MS analysis

##### Mass spectrometry

The mass spectrometric analysis was conducted using a Q-TOF–MS instrument, specifically the Triple TOF 5600 + model from SCIEX (Foster City, CA, USA). The instrument operated in both positive and negative ion modes with an electrospray ionization (ESI) ion source. The detection mode was set as information-dependent acquisition (IDA). Each MS cycle comprised of a single TOF–MS survey scan with an accumulation time of 0.25 s, followed by up to ten IDA product ion scans with an accumulation time of 0.09 s. The mass range for the TOF–MS full scan was set from 50 to 1500 m/z, while for the TOF–MS/MS scan, it was set from 50 to 1250 m/z. The accumulation time for both scans was 0.25 s. Other instrumental parameters included a source temperature of 550 ℃, curtain gas (CUR) maintained at 40 psi, and ion source gas 1 (GS1) and ion source gas 2 (GS2) both set at 50 psi. The ion spray voltage was fixed at 5500 V for the positive mode and -4500 V for the negative mode. The declustering potential (DP) was set at 60 V for the positive mode and -60 V for the negative mode. The collision energy (CE) was set to 40 eV, with a CE spread (CEs) of 20 eV.

##### Liquid chromatography

The chromatographic separation was carried out using an Exion LC™ AD system from SCIEX (Foster City, CA, USA). For sample separation, a Waters Acquity UHPLC HSS T3 column with dimensions of 2.1 mm × 100 mm and a particle size of 1.8 μm was utilized. The flow rate was set at 0.3 mL/min, and the column temperature was maintained at 40 ℃. A gradient elution method was employed using a mobile phase consisting of 0.1% formic acid (A) and acetonitrile (B). The gradient program was configured as follows: from 0 to 5 min, the proportion of solvent B increased from 5 to 20%; from 5 to 10 min, it further increased from 20 to 30%; from 10 to 15 min, it increased from 30 to 43%; from 15 to 20 min, it increased from 43 to 60%; from 20 to 30 min, it increased from 60 to 100%; from 30 to 30.5 min, it decreased from 100 to 5% B; and finally, from 30.5 to 35 min, it was maintained at 5% B. The injection volume for each sample was 1.8 μL.

#### Statistical analysis

The raw data obtained from the AB SCIEX Q-TOF–MS instrument were subjected to data processing using XCMS software (version 3.6.3). This processing step involved extracting information such as retention times, accurate masses, and peak intensities from the raw data, resulting in a data matrix. The generated data matrix was then exported into SIMCA-P 14.1 software (Umetrics, Umea, Sweden) for subsequent multivariate statistical analysis. Differential compounds were identified by a combination of t-test (*p* < 0.05) and fold change (FC > 1.2 or < 0.8) criteria. This screening process allowed the identification of compounds that exhibited statistically significant differences in abundance between the compared groups.

## Results

### Antagonistic test of strains

Experiments to test antagonism were performed with the five strains of nitrogen-fixing bacteria (J1, J2, G4, T16 and T21) (Suppl. Fig. S1). Results showed that the five bacteria did not affect each other's growth at the junction, indicating that these bacteria are not antagonistic to each other and can thus be co-cultured.

### Plant-growth-promotion of combined bacterial agents

In this study, a mixed liquid bacterial agent containing five strains of nitrogen-fixing bacteria (J1, J2, G4, T16, and T21) was prepared to inoculate *A. mongholicus* seeds, which were then sown in the field. Samples of *A. mongholicus* were collected at 30, 60, and 120 days after sowing. Various growth parameters including plant height, root length, and the dry weights of aerial parts and roots were measured at different growth stages and compared with plants that were seeded with distilled water as a control.

**Fig. 1:**
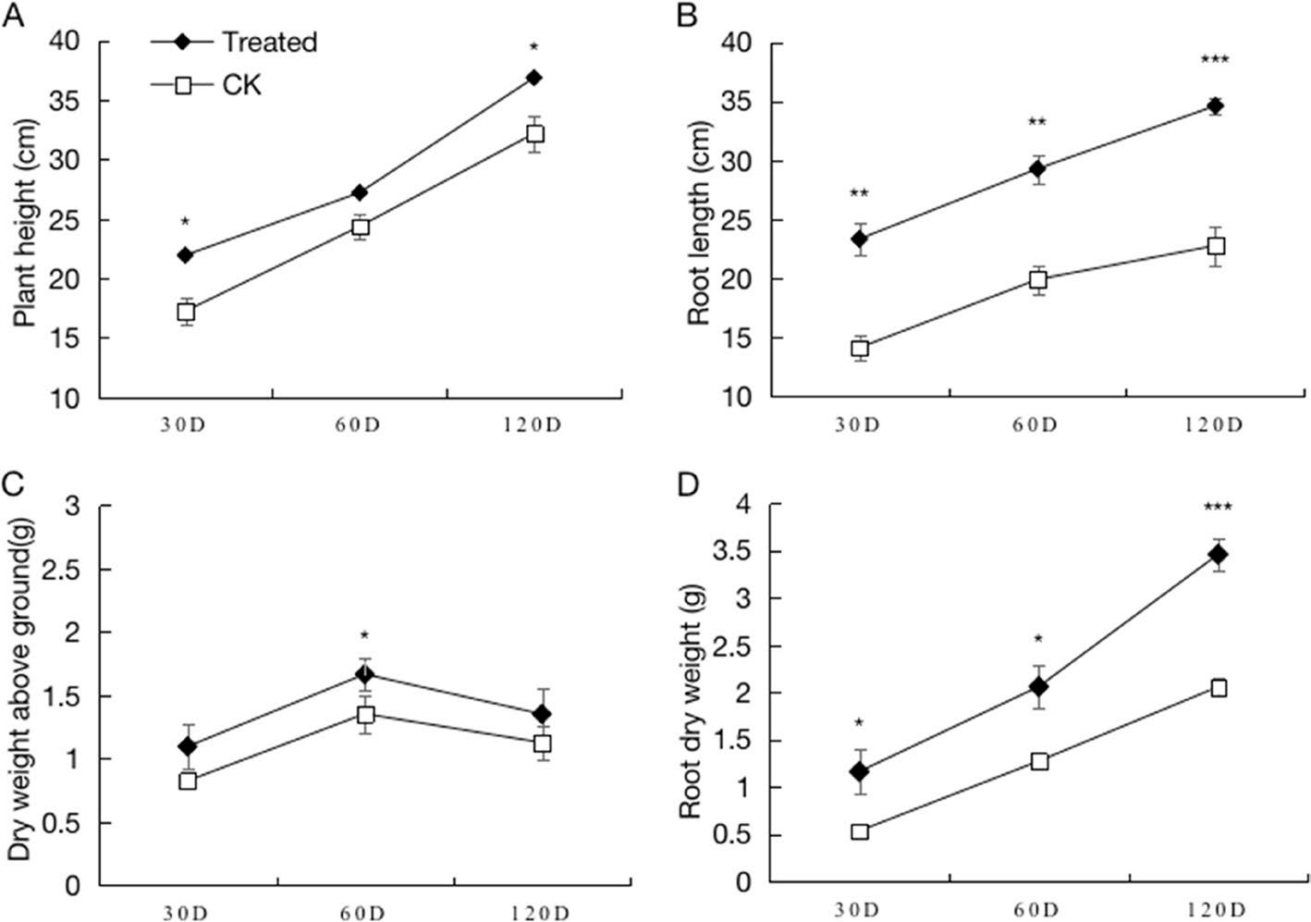
Plant-growth-promotion of *A. mongholicus* treated with bacterial agent. Note: **A** is the effect on the plant height, **B** is the effect on the root length, **C** is the effect on the dry weight of the aerial part of *A. mongholicus,* **D** is the effect on the dry weight of root. *, **, and *** indicate significant differences between bacterial agent treated and control at *p* < 0.05, *p* < 0.01, and *p* < 0.001, respectively

The results showed that the growth of *A. mongholicus* in treatment group was significantly improved (*p* < 0.05) in comparison with the uninoculated control (Fig. 1). Specifically, significant differences in plant height were observed at 30 days and 120 days after sowing when compared with the control group. Root length at 120 days had the most significant difference with an increase of 56.16% compared to the control. At 120 days, the difference in root dry weight was extremely significant, showing a notable increase of 68.06% compared to the control. Moreover, at 60 days, the dry weight of the above-ground parts exhibited a significant increase of 23.18% compared to the control.

During rapid growth stage of *A. mongholicus* (60d after sowing), the number of root nodules in each group was counted (Suppl. Table. S1). Upon comparison with the control group, the treatment group exhibited an average number of root nodules that was more than double. Furthermore, the treatment group had the highest number of nodules per plant among all the studied samples.

### Untargeted metabolomics analysis of *A. mongholicus*

**Fig. 2:**
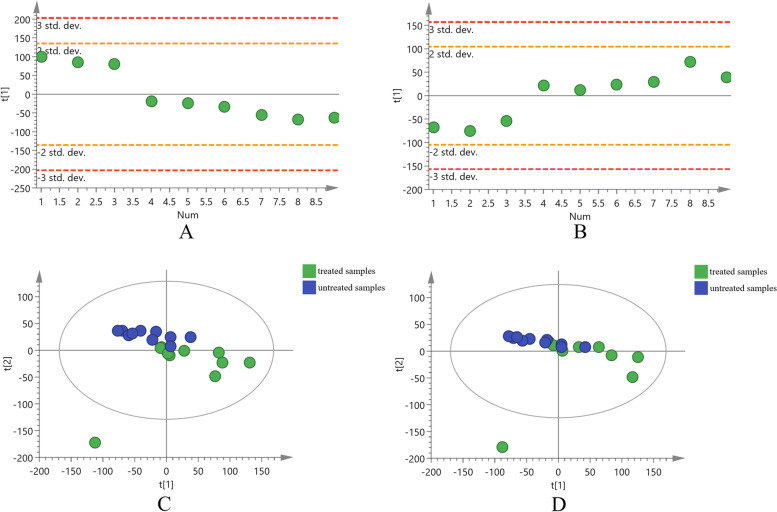
PCA score plots in positive and negative ion modes

Untargeted metabolomics analysis was conducted for root samples of *A. mongholicus* (collected at 120d after sowing) to detect the metabolic difference between groups. The LC–MS data acquired were subjected to XCMS for data processing. In the positive ion mode, a total of 15,893 ions were extracted, while in the negative ion mode, 13,968 ions were extracted. To compare the chemical differences between the nitrogen-fixing bacteria treated and untreated samples, PCA was performed firstly. To evaluate the repeatability of the metabolic profiling, quality control (QC) samples were employed. The PCA score plots of all the QC samples fell within the 2 standard deviations (SD) region (Fig. 2 A and B). This indicates that the LC–MS method utilized in this study exhibited good reproducibility and stability for both the positive and negative ion modes. The PCA score plots (Fig. 2 C and D) demonstrated that although complete separation between the two groups of *A. mongholicus* samples was not achieved, a discernible separation trend could still be observed in both the positive and negative ion modes. Hence, there were indeed chemical differences present between the treated and untreated *A. mongholicus* samples.

With the criteria of *t*-test (*p* < 0.05) and fold change (FC > 1.2 or < 0.8), a total of 26 significantly differential compounds were identified. These compounds consisted of 13 flavonoids, 3 triterpenoid saponins, and 10 other compounds (Table 1). It was interesting that all of these differential compounds were found to be significantly higher in the samples treated with nitrogen-fixing bacteria (Suppl. Fig. S2). It is worth noting that three plant hormones, namely abscisic acid, salicylic acid, and spermidine exhibited higher levels in the treatment group compared to the control group. Although the *p*-values were not statistically significant, their presence in higher quantities in the treatment group suggests a potential influence of the nitrogen-fixing bacteria treatment. (Table 1).

**Table 1:** Differential compounds in untargeted metabolomic results

NO	identification	Mean (treated samples)	Mean (untreated samples)	FC	P	Type
1	Pratensein-O-Glc-Mal	20,468.43	8169.80	2.51	0.0057	isoflavone
2	Pratensein-O-Glc	112,963.45	48,723.59	2.32	0.0147	isoflavone
3	Pratensein-7-O-Glc	27,927.16	10,432.80	2.68	0.0147	isoflavone
4	Formononetin-Glc-Glc-Rha	11,260.88	5997.93	1.88	0.0292	isoflavone
5	Formononetin-7-O-[(E)-but-3-enoyl]-Glc	3541.58	1764.87	2.01	0.0069	isoflavone
6	Dihydroxy-dimethoxy dihydroisoflavone	33,766.89	18,850.38	1.79	0.0411	isoflavone
7	Dihydroxy-dimethoxy dihydroisoflavone-Glc-Mal	7555.65	4403.69	1.72	0.0039	isoflavone
8	5,7,4'-Trihydroxy-3'-methoxyisoflavone-Hex	12,192.25	5069.08	2.41	0.0176	isoflavone
9	Trihydroxy-methoxyisoflavone-Hex	14,278.92	5796.85	2.46	0.0117	isoflavone
10	Isomucronulatol-O-Glc-Ace	9296.62	3841.41	2.42	0.0195	isoflavan
11	Astrapterocarpan-O-Glc-Ace isomer	21,522.24	10,978.78	1.96	0.0028	pterocarpine
12	Astrapterocarpan isomer	12,641.07	4668.99	2.71	0.0377	pterocarpine
13	Dimhydroxyl-trimethoxypterocarpan	2513.98	1354.83	1.86	0.0177	pterocarpine
14	Agroastragaloside IV	32,761.21	14,057.37	2.33	0.0292	triterpenoid saponin
15	Cycloaraloside F	3586.42	1308.45	2.74	0.0310	triterpenoid saponin
16	Sieberoside II	3902.13	1320.80	2.95	0.0413	triterpenoid saponin
17	Tryptophan	31,621.83	17,155.90	1.84	0.0415	amino acid
18	Pipecolinic acid	351,012.02	141,405.49	2.48	0.0161	amino acid
19	Phenylalanine	142,788.93	91,532.78	1.56	0.0227	amino acid
20	Histidine	101,070.32	62,720.93	1.61	0.0265	amino acid
21	Arginine	2,088,759.90	1,075,295.49	1.94	0.0355	amino acid
22	Citric acid	2,366,812.06	1,507,369.52	1.57	0.0294	organic acid
23	Salicylic acid	14,242.08	3035.29	4.69	0.0367	organic acid
24	7-Hydroxycoumarin	2263.73	986.26	2.30	0.0126	coumarin
25	Guanosine	35,593.87	24,993.65	1.42	0.0026	nucleoside
26	Vitamin B2	16,742.79	11,957.08	1.40	0.0444	vitamin
27	Abscisic acid	14,222.22	7476.73	1.90	0.0542	plant hormone
28	spermidine	22,471.78	10,793.11	2.08	0.1455	plant hormone

### Microbial community analysis of rhizosphere soil and root tissue of *A. mongholicus*

For the assessment of bacterial composition, abundance and community diversity in different groups during the growth of *A. mongholicus*, Illumina MiSeq sequencing technology and the Qiime2 microbial analysis platform were employed. A total of 1,968,108 cleaned 16S rRNA reads were obtained from rhizosphere soil samples and 825,820 clean reads from root tissue samples for bacteria community analysis. To analyze the bacterial communities, the pair-end clean reads were firstly merged to tags and then clustered into operational taxonomic units (OTUs) with a 97% similarity threshold. Finally, 2655 OTUs from rhizosphere soil samples and 798 OTUs from root tissue samples were obtained for subsequent analysis.

**Fig. 3:**
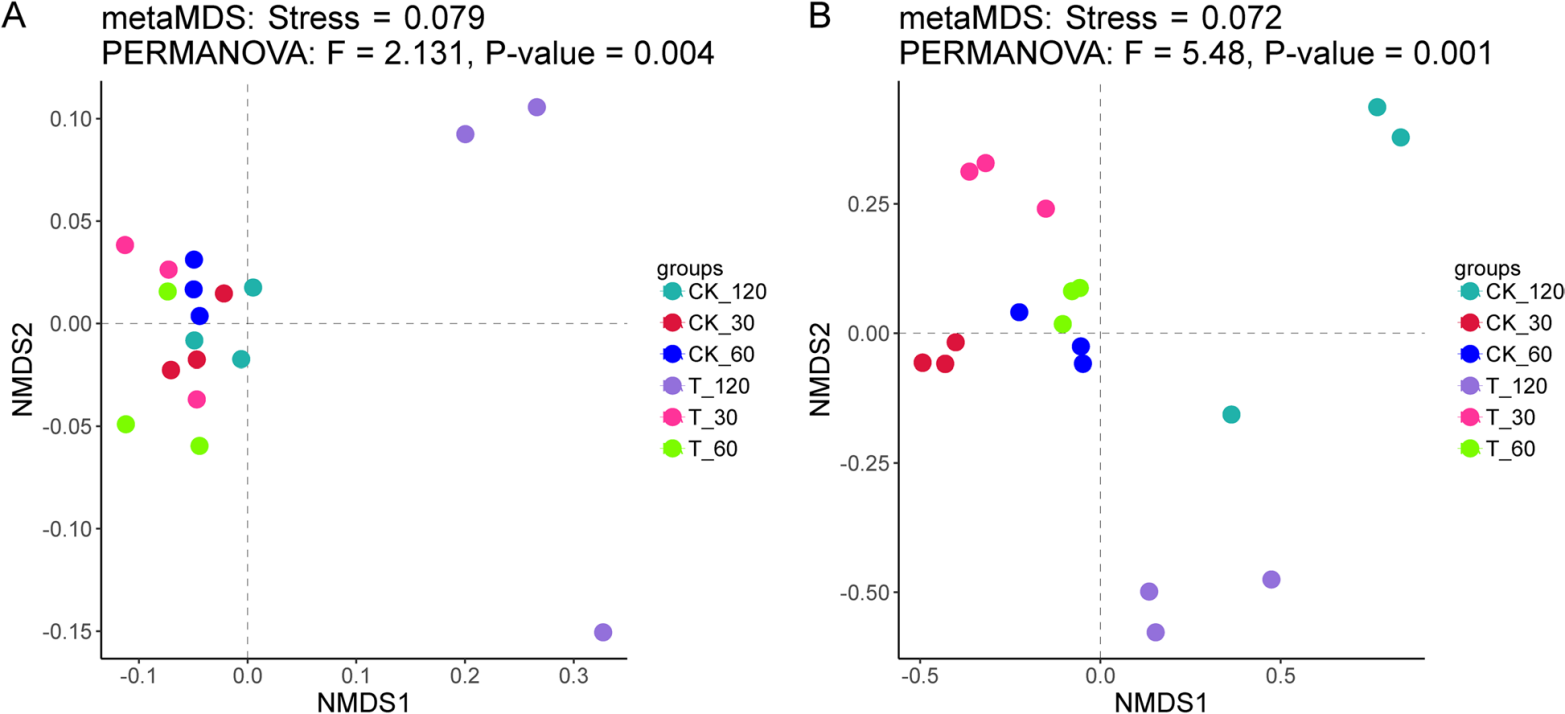
Non-metric multidimensional scaling (NMDS) analysis of bacterial communities based on Bray–Curtis distance for rhizosphere soil (**A**) and root endophytic (**B**) samples at family level

Non-metric multidimensional scaling (NMDS) analysis was performed to assess the bacterial community structure. This analysis aimed to evaluate the similarities between bacterial communities across all groups (Fig. 3). The result showed an overlap in rhizosphere soil bacterial communities between treatment and control groups at 30 and 60 days. However, the bacterial communities of treatment group at 120 days was significantly different compared to other groups (Fig. 3A). For root endophytic bacterial communities, results indicated a clear difference between treatment and control groups at each sampling period (Fig. 3B).

**Fig. 4:**
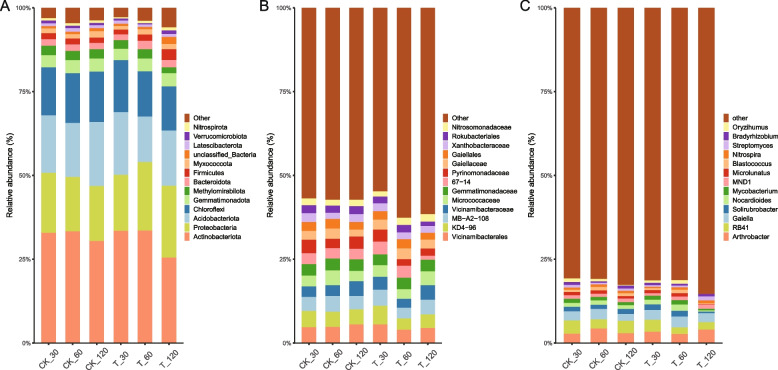
Relative abundance of rhizosphere soil bacteria community at phyla (**A**), family (**B**) and genus (**C**) levels of different groups. Note:X-axis shows the samples collected at 30, 60, and 120 days after sowing (CK: control, T: treated with mixed bacterial agents)

**Fig. 5:**
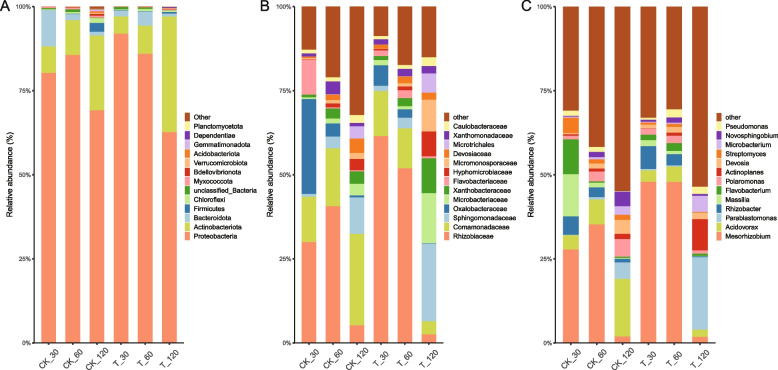
Relative abundance of root endophytic bacteria community at phyla (**A**), family (**B**) and genus (**C**) levels of different groups. Note:X-axis shows the samples collected at 30, 60, and 120 days after sowing (CK: control, T: treated with mixed bacterial agents)

In order to investigate the changes in rhizosphere soil and root endophytic bacterial communities during the growth of *A. mongholicus*, the relative abundance of microbial taxa was assessed at the phylum and family levels between the treatment and control groups (Fig. 4, Fig. 5). For phylum level, Actinobacteriota, Proteobacteria, Acidobacteriota, and Chloroflexi were identified as the predominant phyla in both the treatment and control groups in rhizosphere soil samples throughout the entire growth process of *A. mongholicus* (Fig. 4A). Specifically, Actinobacteriota remained consistently highly abundant throughout the growth period, with an average relative abundance of 32.7%. The relative abundance of Proteobacteria increased during plant growth and reached the highest proportion at 120 days in the treatment group (23.8%). At the family level, Vicinamibacterales, Vicinamibacterace, Micrococcaceae, and Gemmatimonadaceae were the dominant bacteria in all groups (Fig. 4B). At the genus level, Arthrobacter, Gaiella and Solirubrobacter were the dominant bacteria in all groups (Fig. 4C).

Compared with rhizosphere soil, the bacterial microbiota in root endophytic of *A. mongholicus* growth was less diverse (Fig. 5A). *Proteobacteria*, *Actinobacteriota* and *Bacteroidota* were the predominant bacterial phyla. Among them, the proportion of *Proteobacteria* was absolutely dominant with an average level of 78.4%. *Actinobacteriota* increased significantly during plant growth and had the highest proportion in the treatment group at 120 days (35.3%). At the family level, *Rhizobiaceae*, *Comamonadaceae*, *Sphingomonadaceae*, *Oxalobacteraceae,* and *Microbacteriaceae* were the dominant bacteria in root tissue. *Rhizobiaceae* accounted for the highest proportion in root tissue during the growth period of *A. mongholicus.* Furthermore, the relative abundance of *Rhizobiaceae* was found to be significantly higher in the treatment group compared to the control. *Sphingomonadaceae* increased sharply at 120 days in both treatment (22.8%) and control (12.1%), and *Microbacteriaceae* had the same trend (Fig. 5B). At the genus level, Mesorhizobium, Acidovorax, Parablastomonas and Rhizobacter were the predominant bacteria genus. The relative abundance of Mesorhizobium was found to be significantly higher in the treatment group and decreased sharply at 120 days in both group.

**Fig. 6:**
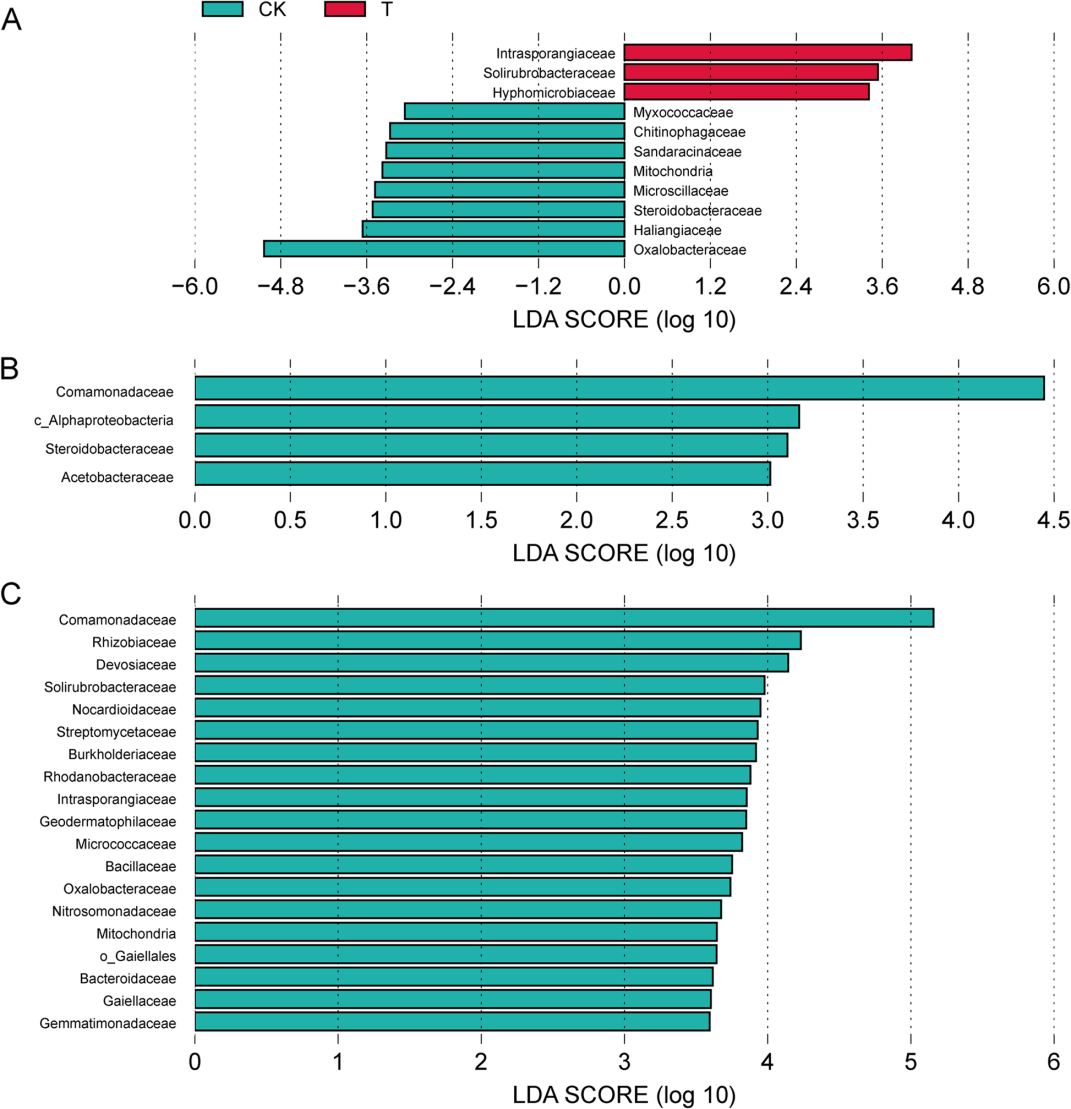
LEfSe analysis of significantly different root endophytic bacteria between treatment and control groups (**A**:30 days, **B**:60 days, **C**:120 days) at family level

**Fig. 7:**
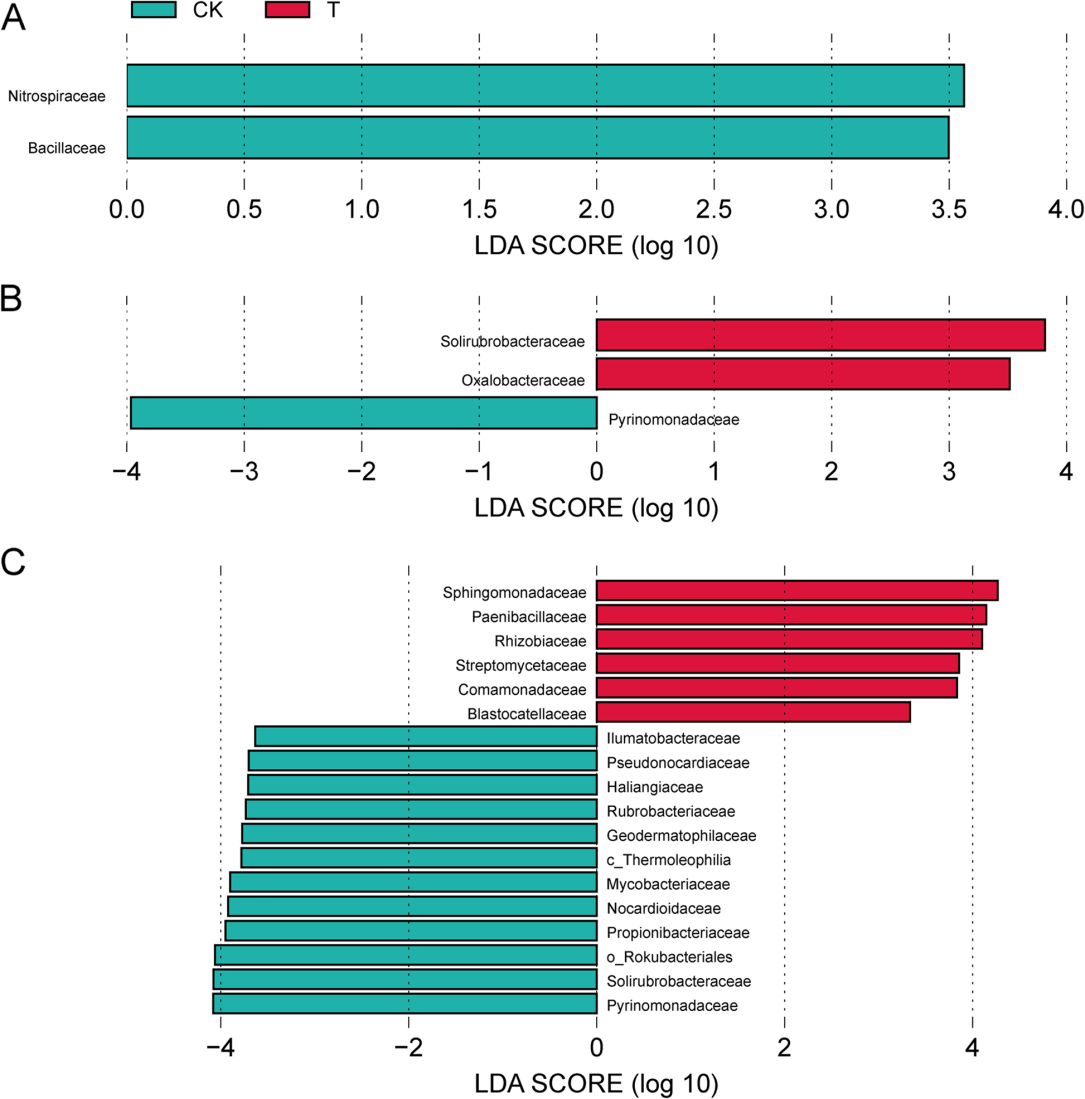
LEfSe analysis of significantly different rhizosphere soil bacteria between treatment and control groups (**A**:30 days, **B**:60 days, **C**:120 days) at family level

In order to detect whether there are statistical differences in the abundance of each strain among the groups, and to screen out these different strains, LEfSe was used to analyze the family level abundance of root endophytic bacterial community (Fig. 6) and rhizosphere bacterial community in each group (Fig. 7). The results revealed that the number of differential bacteria in the root endophytic bacterial community (34) was higher than that of the rhizosphere bacterial community (23), which was consistent with the results of the stacked column chart of relative abundance of bacteria. Among root endophytic bacteria community, treatment group at 30 days had the largest number of differential bacteria, including *Intrasporangiaceae, Solirubrobacteraceae and Hyphomicrobiaceae.* Among rhizosphere bacteria, treatment group at 120 days had the largest number of differential bacteria, among which *Sphingomonadaceae, Paenibacillaceae, Rhizobiaceae, Streptomycetaceae, Comamonadaceae* and *Blastocatellaceae* showed the largest difference. Solirubrobacteraceae and Oxalobacteraceae were also the differential bacteria in the treatment group at 60 days in the rhizosphere bacterial community.

To investigate the primary disparities in bacterial composition across distinct periods, TTest analysis was performed on the predominant bacterial taxa (abundance > 95%) within each group (Suppl. Table S2 for root endophytic bacterial community and Suppl. Table S3 for rhizosphere soil bacterial community). The number of differential bacteria in the root endophytic bacterial community (54) was higher than that of the rhizosphere bacterial community (28), which was consistent with the results of the stacked column chart of bacterial relative abundance and Lefsee analysis. The bacteria with significant increase in abundance in root endophytic of treatment groups were mainly *Intrasporangiaceae, Solirubrobacteraceae, Rhizobiaceae, Mycobacteriaceae* and *Hyphomicrobiaceae* at 30 days, *Flavobacteriaceae* and *Methylophilaceae* at 60 days and *Micromonosporaceae* and *Sphingomonadaceae* at 120 days (Suppl. Table S2). The abundance of *Rhizobiaceae* at 30 days, *Solirubrobacteraceae, Mycobacteriaceae, Ilumatobacteraceae* and *Oxalobacteraceae* at 60 days and *Paenibacillaceae, Streptomycetaceae, Comamonadaceae, Gaiellaceae, Sphingomonadaceae* and *Blastocatellaceae* at 120 days in rhizosphere soil groups were significantly increased after inoculation treatment (Suppl. Table S3).

**Fig. 8:**
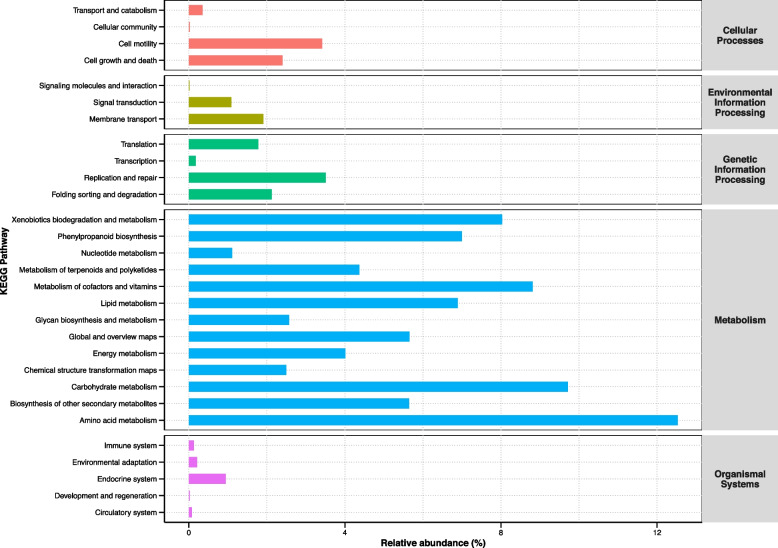
Functional content prediction of significantly different bacteria between treatment and control groups

Functional prediction was conducted using picrust2 for the main abundance differential bacteria (Fig. 8). In the functional prediction of bacteria community exhibiting significant differences in abundance between the treatment and control groups, various metabolic pathways were covered, such as metabolism of terpenoids and polyketides, phenylpropanoid biosynthesis, and the biosynthesis of other secondary metabolites.

The inoculated nitrogen-fixing bacteria strains (J1, J2, G4, T16, and T21) were also detected in the rhizosphere soil samples and root tissue samples (Suppl. Table S4). Results revealed a significant increase in the abundance of *Sinorhizobium sp. T21* in the root tissue after inoculation treatment. This suggests that the inoculation of this particular strain had a notable impact on the bacterial composition in the root tissue. In rhizosphere soil, the abundance of strain *Arthrobacter sp. J2* was observed to be higher than the other inoculated bacteria strains.

## Discussion

In this study, five strains of *A. mongholicus* specific and efficient nitrogen-fixing bacteria, including 2 strains of *Rhizobium* (*Rhizobium sp*. *T16* and *Sinorhizobium sp*. *T21*), 2 strains of *Bacillus* (*Bacillus sp*. *G4* and *Bacillus sp*. *J1*), and one strain of *Arthrobacter* (*Arthrobacter sp*. *J2*), were used to make a combined bacterial agent. It was revealed that the application of the combined nitrogen-fixing bacterial agent had a significant effect on plant-growth-promotion in the field. This finding is consistent with numerous previous research that have demonstrated the beneficial effects of nitrogen-fixing bacteria on plant growth and development. [29–31].

In addition to promoting the yield of *A. mongholicus*, what is more important is whether the bacterial inoculant can improve its medicinal quality, that is, to increase the content of the main bioactive components in the root tissue of *A. mongholicus*. Therefore, untargeted metabolome analysis of the root tissue samples was performed. The results indicated that treatment with the combined bacterial agent significantly increased the accumulation of flavonoid metabolites in the root tissue of *A. mongholicus*. In addition, the accumulation of three triterpenoid saponin metabolites and some amino acids and other substances were also significantly promoted. Thus, the bacterial inoculant showed greater impact on the flavonoids biosynthesis. In contrast to the targeted determination of specific chemical markers, untargeted metabolomic analysis offers a comprehensive assessment of the chemical variances in primary and secondary metabolites. This approach provides a holistic perspective on the impact of bacterial inoculants.

The detected flavonoids in this study were categorized into four groups: isoflavones, isoflavans, flavones, and pterocarpines (Fig. 4). These flavonoids are derived from naringenin, which serves as a precursor. Naringenin is a dihydroflavonoid synthesized from three molecules of malonyl-CoA and one molecule of 4-coumaroyl-CoA. In the biosynthesis pathway of flavonoids in *A. mongholicus*, 4-coumaroyl-CoA, a precursor, is synthesized from phenylalanine through the shikimic acid pathway. Naringenin, derived from 4-coumaroyl-CoA, can undergo enzymatic reactions catalyzed by various enzymes to produce different types of flavonoids. Flavone synthase (FNS) catalyzes the conversion of naringenin into flavones. Isoflavone synthase (IFS) is responsible for transforming naringenin into isoflavones. Flavonol synthase (FLS) and flavanone-3β-hydroxylase (F3H) participate in the conversion of naringenin to flavonols [32–35]. Furthermore, pterocarpines and isoflavans are synthesised from isoflavones through additional enzymatic reactions. These transformations and modifications of flavonoid compounds in *A. mongholicus* involve various enzymes, including hydroxylases, methyltransferases, reductases, and glycosyltransferases. The intricate network of enzymatic reactions and modifications contributes to the formation of structurally diverse flavonoids in *A. mongholicus* [36, 37]. The results of this study revealed that the content of phenylalanine, p-coumaric acid, naringenin, apigenin, daidzin were higher in nitrogen-fixing bacteria treated samples, which was in agreement with the higher isoflavones, isoflavans and pterocarpines in the nitrogen-fixing bacteria treated *A. mongholicus* root samples.

Plant hormones have crucial roles in regulating various aspects of plant biology, such as growth, development, and metabolic processes. In this study, three phytohormones including abscisic acid, salicylic acid, and spermidine, were detected in root tissues of *A. mongholicus,* and their levels were found to be higher in samples treated with nitrogen-fixing bacteria. Previous studies have demonstrated that abscisic acid, salicylic acid, and spermidine are closely associated with plant growth and development, and the accumulation of secondary metabolites [38, 39]. In the case of *Nitraria tangutorum Bobr*, abscisic acid was found to have a positive impact on the defense response of the plant to alkaline stress. This positive regulation of the defense response by abscisic acid can result in the accumulation of specific secondary metabolites, such as flavonoids and anthocyanins [40]. Abscisic acid is a pivotal regulator of drought tolerance in crops and its presence in the rhizosphere has been widely recognized [41]. The active metabolism of Abscisic acid by rhizosphere bacteria suggests its potential involvement in shaping the composition of rhizosphere microbial communities and aiding plants in modulating their interactions with these microorganisms [42]. Salicylic acid contributes to the accumulation of triterpenoid saponin in *Psammosilene tunicoides* [43]. Salicylic acid related exudation signals are essential for enabling both systemic resistance and the plant-mediated formation of a rhizosphere-specific microbiome [44]. Exogenous spermidine has been shown to enhance the total content of flavonoid in lettuce under high temperature stress [45]. The nitrogen-fixing bacteria inoculation treatment resulted in increased accumulation of flavonoids and triterpene saponins in root tissue of *A. mongholicus*. The higher levels of these phytohormones in the treated samples suggest that the presence of nitrogen-fixing bacteria may influence hormone signaling pathways, leading to altered hormone levels and subsequent effects on plant defense and secondary metabolite accumulation. Further research is needed to fully understand the specific mechanisms through which these phytohormones interact with the inoculated bacteria.

Bacteria of the combined bacterial agent (*Rhizobium, Bacillus,* and *Arthrobacter*) primarily interact with the root of the plant and regulate the structure of the microflora in the rhizosphere. This interaction ultimately results in an increase in the biosynthesis of secondary metabolites of the plant. Rhizobium has the function of symbiotic nitrogen fixation, which provides a source of nutrition for host plants. Rhizobium can also induce plant resistance through secondary metabolites of phenols, flavonoids, and plant antitoxins [46]. Besides of nitrogen-fixation ability, *Bacillus* and *Arthrobacter* also have other functions in promoting plant growth. *Bacillus* is capable of directly interacting with the root system of tomato plants and influencing root system responses. It can also induce the release of metabolites from the roots, which promotes the colonization of other microbial species in the rhizosphere soil [47]. The inoculation of nitrogen-fixing bacteria has been shown to have significant benefits as it can increase the yield of the host plant, improve soil fertility, and positively influence the structure of the soil microbial community [48].

The difference in bacterial community structure between combined bacterial agent treatment group and control group was higher in root endophytic bacteria samples than that in rhizosphere soil samples. As plants grow, the composition and abundance of indigenous bacteria in the rhizosphere environment can be affected. Certain bacterial strains may become more dominant while others may be inhibited. This study observed that the dominant bacteria present in the treatment group were distinct from those in the control group, suggesting that the applied treatment influenced the microbial community. *Proteobacteria, Actinobacteriota,* and *Bacteroidota* were found to be the pre-dominant bacterial phyla and *Rhizobiaceae, Comamonadaceae, Sphingomonadaceae, Oxalobacteraceae,* and *Microbacteriaceae* were the dominant bacterial families. The emergence of these dominant bacteria in the treatment group suggests their potential importance in shaping and maintaining the overall structure and functioning of the microbial community. Their presence and activity can contribute to important ecological processes and interactions within the soil ecosystem [49, 50].

The core difference groups of root endophytic bacteria and rhizosphere soil bacteria in different stages after inoculation with combined bacterial agent were explored. The bacteria which abundance increased significantly after being affected by inoculants were found in root tissue (7 bacteria) and rhizosphere soil (11 bacteria), respectively. It is worth mentioning that, in the rhizosphere soil group, under the influence of inoculants, the abundance of Rhizobiaceae during the seedling stage (30 days) and Oxalobacteraceae during the rapid growth phase of A. mongholicus (60 days) was markedly higher compared to the control group. *Rhzobiaceae* can form root nodules in leguminous roots under the control of flavonoids nodulation factors secreted by host root system and promote the absorption of nitrogen by the host [51]. *Oxalobacteraceae* can promote the metabolism of flavonoids in the roots of host plants, and the flavonoids produced by the roots can further promote the enrichment of probiotics bacteria, which in turn promotes the growth of host plant and the acquisition of nitrogen [52]. In root endophytic bacteria, the abundance of *Hypomicrobiaceae* and *Rhzobiaceae* in seedling stage (30 days), *Flavobacteriaceae* in rapid growth period (60 days) and *Micromonosporaceae* in defoliation stage (120 days) were affected by the gain of the inoculant. *Hypomicrobiaceae* and *Rhzobiaceae* belong to the order *Rhizobiaceae* and have the function of symbiotic nitrogen fixation, providing nutrition sources for host plants [51]. *Micromonosporaceae* belongs to *Actinomycetes*, which has the function of decomposing some organic substances, such as cellulose, chitin, and xylan, and can produce a variety of antibiotics. It has been reported that a variety of Micromonosporaceae isolated from plant root tissue can promote the synthesis of plant terpenes, isoflavones, flavonoids, aminoglycoside, and alkaloids [53]. It is speculated that these core differential bacteria may have a significant impact on the regulation of secondary metabolism of the host plant, leading to the accumulation of flavonoids and saponins in the root.

This study provides novel insights into the multifaceted effects of inoculating mixed nitrogen-fixing bacteria on host plant growth of *A. mongholicus*. It demonstrates that the influence of the inoculation is diverse and encompasses various mechanisms. Firstly, the mixed bacteria were capable of symbiotic and non-symbiotic nitrogen fixation, which contributes to the availability of nitrogen for the plants. This, in turn, promotes nutrient absorption by the plants and supports their overall growth. Additionally, the inoculation of mixed bacteria was found to regulate phytohormones within the host plants. This hormonal regulation can have significant impacts on plant growth and development. Furthermore, the inoculation was found to promote the synthesis of secondary metabolites. These secondary metabolites, such as flavonoids and saponins, are known to play important roles in enhancing the tolerance of plants to stress conditions. Overall, the findings suggest that the inoculation of mixed bacteria has a comprehensive impact on host plant growth. These effects ultimately promote the accumulation of key bioactive components, such as flavonoids and saponins, in *A. mongholicus.*

## Conclusions

In this study, five strains of *A. mongholicus* specific and efficient nitrogen-fixing bacteria were made into a combined bacteria agent and was applied in the planting of *A. mongholicus* in the field. The application of the combined bacterial agent had several positive effects. It promoted plant growth and enhanced the accumulation of the main medicinal bioactive components in *A. mongholicus*. These effects were attributed to the promotion of nitrogen-fixing activity, the regulation of phytohormones such as abscisic acid, salicylic acid, and spermidine, and the modulation of the root endophytic and rhizosphere bacterial communities which may potentially lead to beneficial interactions and ecological effects. *Rhzobiaceae, Oxalobacteraceae, Micromonosporaceae* and *Hypomicrobiaceae* are supposed to have a significant role in these process*.* These findings provide a basis for application of such nitrogen-fixing bacteria as a beneficial PGRP agent for the cultivation of *A. mongholicus*.

### Supplementary Information

Supplementary Material 1.

## Data Availability

The data presented in this study are deposited in NCBI (accession numbers: SRX23334251-SRX23334286 and PRJNA1066998). Further inquiries can be directed to the corresponding author.

## References

[1] James MMYTC-HCKCAE, Ko JKS (2007). Astragalus saponins induce growth inhibition and apoptosis in human colon cancer cells and tumor xenograft. Carcinogenesis.

[2] Yang B, Xiao B, Sun T (2013). Antitumor and immunomodulatory activity of astragalus membranaceus polysaccharides in h22 tumor-bearing mice. Int J Biol Macromol.

[3] Zhou R, Chen H, Chen J, Chen X, Wen Y, Xu L (2018). Extract from astragalus membranaceus inhibit breast cancer cells proliferation via pi3k/akt/mtor signaling pathway. BMC Complement Altern Med.

[4] Rahman, K.M.A., and Zhang, D. 2018. Effects of Fertilizer Broadcasting on the Excessive Use of Inorganic Fertilizers and Environmental Sustainability. Sustainability. 10(3). 10.3390/su10030759.

[5] Tabassum B, Khan A, Tariq M, Ramzan M, Aaliya K (2017). Bottlenecks in commercialisation and future prospects of pgpr. Appl Soil Ecol.

[6] Glick BR (2014). Bacteria with ACC deaminase can promote plant growth and help to feed the world. Microbiol Res.

[7] Faist H, Trognitz F, Antonielli L, Symanczik S, White PJ, Sessitsch A (2023). Potato root-associated microbiomes adapt to combined water and nutrient limitation and have a plant genotype-specific role for plant stress mitigation. Environmental Microbiome.

[8] Trivedi P, Leach JE, Tringe SG, Sa T, Singh BK (2020). Plant-microbiome interactions: from community assembly to plant health. Nat Rev Microbiol.

[9] Bhardwaj D, Ansari MW, Sahoo RK, Tuteja N (2014). Biofertilizers function as key player in sustainable agriculture by improving soil fertility, plant tolerance and crop productivity. Microb Cell Fact.

[10] Ahmed M, Rauf M, Mukhtar Z, Saeed NA (2017). Excessive use of nitrogenous fertilizers: an unawareness causing serious threats to environment and human health. Environ Sci Pollut Res.

[11] Ambrosini A, Passaglia LMP (2017). Plant Growth-Promoting Bacteria (PGPB): Isolation and Screening of PGP Activities. Curr Protoc Plant Biol.

[12] Mei, X., Wang, S., Zhang, L., et al. 2022.Widely targeted metabolomics analysis revealed components change regularity of *Salvia miltiorrhiza Bunge* after post-harvest drying under different temperature. Industrial Crops and Products. 188(115638). 10.1016/j.indcrop.2022.115638.

[13] Zhang, Q,. Li, B., Chen, Q., et al. 2021. Non-targeted metabolomic analysis of the variations in the metabolites of two genotypes of Glycyrrhiza uralensis Fisch. under drought stress. Industrial Crops and Products. 176 (114402). 10.1016/j.indcrop.2021.114402.

[14] Olanrewaju, O.S., Glick, B.R., and Baba Lola, O.O. Mechanisms of action of plant growth promoting bacteria. *World Journal of Microbiology & Biotechnology* 2017, 33(11), 197. 10.1007/s11274-017-2364-9. 10.1007/s11274-017-2364-9 PMC5686270 28986676

[15] Taurian, T., Soledad Anzuay, M., Luduena, L.M., Angelini, J.G., Munoz, V., Valetti, L., et al. 2013. Effects of single and co-inoculation with native phosphate solubilising strain Pantoea sp J49 and the symbiotic nitrogen fixing bacterium Bradyrhizobium sp SEMIA 6144 on peanut (Arachis hypogaea L.) growth. Symbiosis. 59(2)**,** 77–85. 10.1007/s13199-012-0193-z.

[16] Beneduzi, A., Ambrosini, A., and Passaglia, L.M. 2012. Plant growth-promoting rhizobacteria (PGPR): Their potential as antagonists and biocontrol agents. Genet Mol Biol. 35 (4 (suppl)), 1044–1051. 10.1590/s1415-47572012000600020. 10.1590/s1415-47572012000600020 PMC3571425 23411488

[17] Kwak MJ, Kong HG, Choi K, Kwon SK, Song JY, Lee J (2018). Rhizosphere microbiome structure alters to enable wilt resistance in tomato. Nat biotechnol.

[18] Olanrewaju OS, Glick BR, Baba Lola OO (2017). Mechanisms of action of plant growth promoting bacteria. World J Microbiol Biotechnol.

[19] Pang Z, Chen J, Wang T, Gao C, Li Z, Guo L (2021). Linking plant secondary metabolites and plant microbiomes: a review. Front Plant Sci.

[20] Trivedi P, Leach JE, Tringe SG, Sa T, Singh BK (2020). Plant-microbiome interactions: from community assembly to plant health[J]. Nat Rev Microbiol.

[21] Hu L, Robert CAM, Cadot S, Zhang X, Ye M, Li B (2018). Root exudate metabolites drive plant-soil feedbacks on growth and defence by shaping the rhizosphere microbiota. Nat Commun.

[22] Li Y, Kong D, Fu Y, Sussman MR, Wu H (2020). The effect of developmental and environmental factors on secondary metabolites in medicinal plants. Plant Physiol Bioch.

[23] Liang JP, Xue ZQ, Yang ZY (2021). Effects of microbial organic fertilizers on Astragalus membranaceus growth and rhizosphere microbial community. Ann Microbiol.

[24] Shi Z, Guo X, Lei Z (2023). Screening of high-efficiency nitrogen-fixing bacteria from the traditional Chinese medicine plant Astragalus mongolicus and its effect on plant growth promotion and bacterial communities in the rhizosphere. BMC Microbiol.

[25] Bolyen E, Rideout JR, Dillon MR, Bokulich NA, Abnet CC, Al-Ghalith GA (2019). Reproducible, interactive, scalable and extensible microbiome data science using QIIME 2. Nat Biotechnol.

[26] Segata N, Izard J, Waldron L, Gevers D, Miropolsky L, Garrett WS, Huttenhower C (2011). Metagenomic biomarker discovery and explanation. Genome Biol.

[27] Douglas GM, Maffei VJ, Zaneveld JR (2020). PICRUSt2 for prediction of metagenome functions. Nat Biotechnol.

[28] DeSantis TZ, Hugenholtz P, Larsen N, Rojas M, Brodie EL, Keller K, Huber T, Dalevi D, Hu P, Andersen GL (2006). Greengenes, a chimera-checked 16s rrna gene database and workbench compatible with arb. Appl Environ Microbiol.

[29] Chi F, Yang P, Han F, Jing Y, Shen S (2010). Proteomic analysis of rice seedlings infected by sinorhizobium meliloti 1021. Proteomics.

[30] Gage DJ (2004). Infection and invasion of roots by symbiotic, nitrogen-fixing rhizobia during nodulation of temperate legumes. Microbiol Mol Biol Rev.

[31] Gopalakrishnan, S., Sathya, A., Vijayabharathi, R., Varshney, R. K., Gowda, C. L. L., and Krishnamurthy, L. 2015. Plant growth promoting rhizobia: Challenges and opportunities. 3 Biotech 5, 355–377. 10.1007/s13205-014-0241-x. 10.1007/s13205-014-0241-x PMC4522733 28324544

[32] Li C, Bai Y, Li S (2012). Cloning, Characterization, and Activity Analysis of a Flavonol Synthase Gene FtFLS1 and Its Association with Flavonoid Content in Tartary Buckwheat. J Agric Food Chem.

[33] Yao P, Huang Y, Dong Q (2020). FtMYB6, a Light-Induced SG7 R2R3-MYB Transcription Factor, Promotes Flavonol Biosynthesis in Tartary Buckwheat (*Fagopyrum tataricum*). J Agric Food Chem.

[34] Zhang L, Li X, Ma B, Gao Q, Du H, Han Y (2017). The tartary buckwheat genome provides insightsinto rutin biosynthesis and abiotic stresstolerance. Mol Plant.

[35] Zhao Q, Zhang Y, Wang G, Hill L, Weng JK, Chen XY (2016). A specialized flavone biosynthetic pathway has evolved in the medicinal plant, scutellaria baicalensis. Sci Adv.

[36] Pandey, R. P. , Parajuli, P. , Koffas, M. A. G. , & Sohng, J. K. 2016. Microbial production of natural and non-natural flavonoids: pathway engineering, directed evolution and systems/synthetic biology. Biotechnology Advances, 634–662. 10.1016/j.biotechadv.2016.02.012. 10.1016/j.biotechadv.2016.02.012 26946281

[37] Zhang F, Zhang X, Luo Y (2022). Biosynthetic mechanisms of isoflavone accumulation affected by different growth patterns in Astragalus mongholicus products. BMC Plant Biol.

[38] Hayat S, Hasan SA, Fariduddin Q, Ahmad A (2008). Growth of tomato (Lycopersicon esculentum) in response to salicylic acid under water stress. Journal of Plant Interactions.

[39] Kanchan V, Neha U, Nitin K, Gaurav Y, Jaspreet S, Mishra RK (2017). Abscisic acid signaling and abiotic stress tolerance in plants: a review on current knowledge and future prospects. Front Plant Sci.

[40] Zhang, J., Cheng, K., et al. 2023. Exogenous abscisic acid and sodium nitroprusside regulate flavonoid biosynthesis and photosynthesis of Nitraria tangutorum Bobr in alkali stress. Front. Plant Sci., 14. 10.3389/fpls.2023.1118984. 10.3389/fpls.2023.1118984 PMC10057120 37008502

[41] Sah SK, Reddy KR, Li J (2016). Abscisic Acid and Abiotic Stress Tolerance in Crop Plants. Front Plant Sci.

[42] Belimov AA, Dodd IC, Safronova VI, Dumova VA, Shaposhnikov AI, Ladatko AG, Davies WJ (2014). Abscisic acid metabolizing rhizobacteria decrease ABA concentrations in planta and alter plant growth. Plant Physiol Biochem.

[43] Su, L. , Li, S. , Qiu, H. , Wang, H. , & Zhang, Z. 2021. Full-length transcriptome analyses of genes involved in triterpenoid saponin biosynthesis of psammosilene tunicoides hairy root cultures with exogenous salicylic acid. Frontiers in Genetics*,* 12. 10.3389/fgene.2021.657060. 10.3389/fgene.2021.657060 PMC8039526 33854529

[44] Lebeis SL, Paredes SH, Lundberg DS, Breakfield N, Gehring J, McDonald M, Malfatti S, Glavina del Rio T, Jones CD, Tringe SG, Dangl JL (2015). Salicylic acid modulates colonization of the root microbiome by specific bacterial taxa. Science.

[45] Sun W, Hao J, Fan S, Liu C, Han Y (2022). Transcriptome and Metabolome Analysis Revealed That Exogenous Spermidine-Modulated Flavone Enhances the Heat Tolerance of Lettuce. Antioxidants.

[46] Arumugam, R. , Rajasekaran, S. , & Nagarajan, S. M. 2010. Response of arbuscular mycorrhizal fungi and rhizobium inoculation on growth and chlorophyll content of vigna unguiculata (L) walp var. pusa 151. Journal of Applied Sciences & Environmental Management, 14 (4) 113 - 115. 10.4314/jasem.v14i4.63282.

[47] Korenblum E, Dong Y, Szymanski J, Panda S, Jozwiak A, Massalha H (2020). Rhizosphere microbiome mediates systemic root metabolite exudation by root-to-root signaling. Proc Natl Acad Sci U S A.

[48] Cordeiro C, Echer FR (2019). Interactive Effects of Nitrogen-Fixing Bacteria Inoculation and Nitrogen Fertilization on Soybean Yield in Unfavorable Edaphoclimatic Environments. Sci Rep.

[49] Dal Cortivo, C., Ferrari, M., Visioli, G., Lauro, M., Fornasier, F., Barion, G., et al. 2020. Effects of seed-applied biofertilizers on rhizosphere biodiversity and growth of common wheat (Triticum aestivum L.) in the field. Front. Plant Sci. 11:72. 10.3389/fpls.2020.00072. 10.3389/fpls.2020.00072 PMC7054350 32174929

[50] Wei Z, Gu Y, Friman V-P, Kowalchuk GA, Xu Y, Shen Q, et al. 2019. Initial soil microbiome composition and functioning predetermine future plant health. Sci Adv. 5:eaaw0759. 10.1126/sciadv.aaw0759. 10.1126/sciadv.aaw0759 PMC6760924 31579818

[51] Hemmerle L, Maier BA, Bortfeld-Miller M, Ryback B, Gbelein CG, Ackermann M (2022). Dynamic character displacement among a pair of bacterial phyllosphere commensals in situ. Nat Commun.

[52] Yu P , He X , Baer M ,et al. 2023. Plant flavones enrich rhizosphere Oxalobacteraceae to improve maize performance under nitrogen deprivation. Nature Plants*.* 6(16). 10.1038/s41477-021-00897-y. 10.1038/s41477-021-00897-y 33833418

[53] Li Z, Xiong K, Wen W, Li L, Xu D (2023). Functional Endophytes Regulating Plant Secondary Metabolism: Current Status, Prospects and Applications. Int J Mol Sci.

