# Supplementary material for: Nitrogen-fixing bacteria promote growth and bioactive components accumulation of Astragalus mongholicus by regulating plant metabolism and rhizosphere microbiota
Source: BMC Microbiol. 2024 Jul 15;24:261. doi: 10.1186/s12866-024-03409-y (PMC11247893; doi:10.1186/s12866-024-03409-y)
Supplement: Supplementary file 1 — Supplementary Material 1. [file 12866_2024_3409_MOESM1_ESM.zip › Supplementary material.docx]

**Supplementary material**

Suppl. Table S1 Root nodules count in each group

| sample | Number of root nodules | |
| --- | --- | --- |
|  | Control | Treated |
| 1 | 1 | 7 |
| 2 | 1 | 2 |
| 3 | 1 | 2 |
| 4 | 0 | 0 |
| 5 | 2 | 4 |
| 6 | 0 | 1 |
| 7 | 4 | 3 |
| 8 | 1 | 2 |
| 9 | 2 | 3 |
| 10 | 1 | 4 |
| average | 1.3 | 2.8 |
| FC | 2.153846154 | |
| *p* | 0.030571898 | |

Suppl. Table S2. Analysis of root endophytic bacteria at family level.

| Sample date | Taxonomy | Treatment | Control | FC | *P* | VIP |
| --- | --- | --- | --- | --- | --- | --- |
| 30d | Steroidobacteraceae | 0.000199555 | 0.001103234 | 0.180881801 | 0.005099567 | 1.76026546 |
|  | Mitochondria | 0.000271476 | 0.001055041 | 0.257313262 | 0.011951415 | 1.664570194 |
|  | Haliangiaceae | 4.99E-05 | 0.000590393 | 0.084463232 | 0.012987831 | 1.485266604 |
|  | Microscillaceae | 8.31E-05 | 0.000746761 | 0.1112558 | 0.063560098 | 1.245862806 |
|  | Intrasporangiaceae | 0.000624586 | 0.000144589 | 4.319736819 | 0.074795728 | 1.24764423 |
|  | Pseudomonadaceae | 0.005716797 | 0.01534038 | 0.372663314 | 0.089766953 | 1.283023612 |
|  | Micromonosporaceae | 0.001075454 | 0.00424491 | 0.253351436 | 0.105111319 | 1.124768935 |
|  | Solirubrobacteraceae | 0.000811092 | 0.000272136 | 2.980468798 | 0.122667229 | 1.057057445 |
|  | Chitinophagaceae | 4.99E-05 | 0.000893264 | 0.055824985 | 0.141625784 | 1.001310682 |
|  | Myxococcaceae | 2.40E-05 | 0.000910441 | 0.026396755 | 0.142719789 | 1.021953188 |
|  | Rhizobiaceae | 0.614387357 | 0.299211854 | 2.053352327 | 0.143773384 | 1.020663394 |
|  | Methylophilaceae | 0.001343417 | 0.003313196 | 0.405474696 | 0.15245423 | 0.996825684 |
|  | Flavobacteriaceae | 0.016200603 | 0.103686156 | 0.156246538 | 0.156795559 | 0.812483948 |
|  | Mycobacteriaceae | 0.003387207 | 0.000541786 | 6.251929997 | 0.166041335 | 0.843555968 |
|  | Hyphomicrobiaceae | 0.004816777 | 0.000862808 | 5.582673514 | 0.17614639 | 0.774376751 |
| 60d | c_Alphaproteobacteria | 0.000213525 | 0.001706197 | 0.125146532 | 0.008317471 | 1.885080893 |
|  | Steroidobacteraceae | 0.001825082 | 0.003503697 | 0.520901811 | 0.065979447 | 1.621272732 |
|  | Acetobacteraceae | 0.000426205 | 0.00139226 | 0.306124339 | 0.074607448 | 1.595682508 |
|  | Flavobacteriaceae | 0.023073381 | 0.003772205 | 6.116682763 | 0.096969391 | 1.41777491 |
|  | Beijerinckiaceae | 0.015466176 | 0.026504875 | 0.583521938 | 0.109764212 | 1.381924366 |
|  | Micrococcaceae | 0.00278685 | 0.004973135 | 0.560381014 | 0.119595173 | 1.379564928 |
|  | Rhodanobacteraceae | 0.004000208 | 0.008637568 | 0.463117456 | 0.126725408 | 1.335524162 |
|  | c_Acidimicrobiia | 0.000449046 | 0.001223514 | 0.367013735 | 0.133191851 | 1.288499624 |
|  | Erwiniaceae | 0.000335479 | 0.003185286 | 0.105321318 | 0.136465563 | 1.152331015 |
|  | Actinospicaceae | 0.000115211 | 0.001595413 | 0.072213724 | 0.144639649 | 1.146792906 |
|  | Comamonadaceae | 0.119216014 | 0.172045724 | 0.692932153 | 0.169825009 | 1.110208773 |
|  | Methylophilaceae | 0.002540878 | 0.001510661 | 1.681963966 | 0.185297668 | 1.051349569 |
|  | Microbacteriaceae | 0.00902464 | 0.014808271 | 0.609432366 | 0.219375312 | 1.045774391 |
|  | Rhizobiales_Incertae_Sedis | 0.003285727 | 0.004647553 | 0.706979865 | 0.226636177 | 0.992147859 |
| 120d | Micrococcaceae | 0.000296421 | 0.008437288 | 0.035132211 | 0.001114449 | 1.062957925 |
|  | Rhizobiaceae | 0.02479376 | 0.051654319 | 0.479993936 | 0.004404986 | 1.099509347 |
|  | Comamonadaceae | 0.038869194 | 0.271982735 | 0.142910518 | 0.01178417 | 1.053766905 |
|  | Geodermatophilaceae | 0.000131228 | 0.00276621 | 0.047439717 | 0.022257707 | 1.05507329 |
|  | Mitochondria | 0.002616728 | 0.004818657 | 0.543040918 | 0.024085993 | 1.155301651 |
|  | Bacillaceae | 0.000369138 | 0.008701389 | 0.042422877 | 0.028882269 | 0.943586078 |
|  | Nocardioidaceae | 0.004656565 | 0.012364874 | 0.376596216 | 0.033776819 | 1.057288658 |
|  | Streptomycetaceae | 0.002416644 | 0.014723708 | 0.164132 823 | 0.035277779 | 1.063126358 |
|  | Nitrosomonadaceae | 0.000380132 | 0.005480708 | 0.069358153 | 0.041959008 | 0.976848698 |
|  | Solirubrobacteraceae | 0.001625031 | 0.010329588 | 0.157318092 | 0.044228183 | 0.991067014 |
|  | Burkholderiaceae | 0.004001294 | 0.013715727 | 0.291730388 | 0.051190723 | 1.149044581 |
|  | Gaiellaceae | 0.000203946 | 0.005442489 | 0.037472858 | 0.065959087 | 0.947485179 |
|  | Devosiaceae | 0.020649929 | 0.042630477 | 0.484393563 | 0.081928727 | 1.133391155 |
|  | o_Gaiellales | 0.000242101 | 0.006798349 | 0.035611673 | 0.085968843 | 0.91287486 |
|  | Intrasporangiaceae | 4.23E-05 | 0.008679506 | 0.004878826 | 0.091349171 | 0.950856607 |
|  | Pseudonocardiaceae | 0.00277272 | 0.016990121 | 0.163196 | 0.100181348 | 1.065150753 |
|  | Beijerinckiaceae | 0.009220588 | 0.021370522 | 0.431462909 | 0.102534877 | 1.098293616 |
|  | Bacteroidaceae | 0 | 0.004450254 | 0 | 0.113500571 | 0.839608389 |
|  | Gemmatimonadaceae | 2.55E-05 | 0.004316857 | 0.005913361 | 0.117875734 | 0.979040437 |
|  | Micromonosporaceae | 0.094758673 | 0.018152413 | 5.220169646 | 0.13772717 | 1.40591685 |
|  | c_Alphaproteobacteria | 0.019195041 | 0.010822771 | 1.773579127 | 0.170028915 | 1.281530391 |
|  | c_Acidimicrobiia | 0.001000025 | 0.002866793 | 0.348830622 | 0.171134088 | 1.08976117 |
|  | Oxalobacteraceae | 0.001434147 | 0.0056702 | 0.252927132 | 0.175766922 | 1.056637781 |
|  | Sphingomonadaceae | 0.230960083 | 0.108662092 | 2.125489014 | 0.186151505 | 1.312934508 |

###

### Suppl. Table S3. Analysis of rhizosphere soil bacteria at family level.

| Sample date | Taxonomy | Treatment | Control | FC | *P* | VIP |
| --- | --- | --- | --- | --- | --- | --- |
| 30d | Bacillaceae | 0.005977555 | 0.007542066 | 0.792562011 | 0.139770996 | 1.782820571 |
|  | Rhizobiaceae | 0.018621834 | 0.006157546 | 3.024229936 | 0.00825636 | 1.315573315 |
| 60d | Solirubrobacteraceae | 0.020888464 | 0.016074037 | 1.299515751 | 0.01882055 | 1.834093242 |
|  | Micrococcaceae | 0.02859535 | 0.044187692 | 0.647133808 | 0.079856155 | 1.828631765 |
|  | Mycobacteriaceae | 0.013312543 | 0.010289266 | 1.293828195 | 0.137291805 | 1.54321876 |
|  | Ilumatobacteraceae | 0.014116572 | 0.011037952 | 1.278912342 | 0.158860741 | 1.585171221 |
|  | Oxalobacteraceae | 0.010943988 | 0.007510539 | 1.457150844 | 0.166140865 | 1.444448818 |
|  | Pyrinomonadaceae | 0.019593704 | 0.027873326 | 0.702955352 | 0.19059476 | 1.471633404 |
| 120d | Nocardioidaceae | 0.008144237 | 0.014942234 | 0.545048109 | 0.000600887 | 1.400262671 |
|  | Propionibacteriaceae | 0.002009827 | 0.007948345 | 0.252861057 | 0.000843161 | 1.379919966 |
|  | Mycobacteriaceae | 0.004421555 | 0.008912562 | 0.49610371 | 0.000844637 | 1.375938301 |
|  | c_Thermoleophilia | 0.003003374 | 0.0064636 | 0.464659572 | 0.003474919 | 1.372985666 |
|  | Haliangiaceae | 0.003328531 | 0.007187566 | 0.463095763 | 0.004708247 | 1.377254569 |
|  | Paenibacillaceae | 0.016400599 | 0.003663462 | 4.47680333 | 0.005313972 | 1.369543033 |
|  | o_Rokubacteriales | 0.013260545 | 0.023847861 | 0.55604756 | 0.006688554 | 1.362849069 |
|  | Solirubrobacteraceae | 0.00771487 | 0.019124429 | 0.403403939 | 0.006848078 | 1.344480374 |
|  | Ilumatobacteraceae | 0.006988992 | 0.011015396 | 0.634474859 | 0.015516691 | 1.313038735 |
|  | Geodermatophilaceae | 0.003080356 | 0.008980158 | 0.343018064 | 0.017704817 | 1.281657276 |
|  | Streptomycetaceae | 0.012415439 | 0.006388024 | 1.943549277 | 0.018081253 | 1.273399845 |
|  | Pseudonocardiaceae | 0.004144959 | 0.008639923 | 0.479744924 | 0.023652152 | 1.260100779 |
|  | Rubrobacteriaceae | 0.002198822 | 0.007208312 | 0.305039712 | 0.034156359 | 1.249011387 |
|  | Comamonadaceae | 0.012912837 | 0.007350077 | 1.756830167 | 0.047749501 | 1.238310767 |
|  | Pyrinomonadaceae | 0.021858913 | 0.036840604 | 0.593337533 | 0.067601232 | 1.21814443 |
|  | Roseiflexaceae | 0.008693839 | 0.013941003 | 0.623616454 | 0.067628406 | 1.224497534 |
|  | Gaiellaceae | 0.026606815 | 0.020474264 | 1.299524843 | 0.087963653 | 1.221139173 |
|  | Micromonosporaceae | 0.008369675 | 0.011406591 | 0.733757818 | 0.141424158 | 1.177339872 |
|  | Sphingomonadaceae | 0.028056256 | 0.010708124 | 2.620090643 | 0.160986962 | 1.085761746 |
|  | Blastocatellaceae | 0.0062017 | 0.004269539 | 1.452545644 | 0.169637048 | 1.05635039 |

Suppl. Table S4. Absolute abundance of inoculated bacteria strains.

| group | taxonomy | T_30d | CK_30d | T_60d | CK_60d | T_120d | CK_120d |
| --- | --- | --- | --- | --- | --- | --- | --- |
| Root tissue | G4 | 0 | 0 | 1 | 0 | 1 | 0 |
|  | J2 | 17±5 | 7±2 | 26±4 | 7±2 | 25±6 | 3±1 |
|  | T21 | 1452±127 | 113±23 | 284±28 | 127±21 | 44±7 | 1 |
|  | T16 | 32±6 | 0 | 69±12 | 2±1 | 17±3 | 1 |
|  | J1 | 1 | 1 | 0 | 0 | 2±1 | 0 |
| Rhizosphere soil | G4 | 25±7 | 1 | 14±5 | 1 | 1 | 2 |
|  | J2 | 1402±361 | 1118±85 | 1159±169 | 984±170 | 1605±336 | 1217±213 |
|  | T21 | 151±34 | 32±11 | 120±27 | 57±16 | 107±13 | 24±7 |
|  | T16 | 6±3 | 6±3 | 16±4 | 4±1 | 4±2 | 7±2 |
|  | J1 | 34±9 | 19±7 | 65±17 | 42±8 | 46±12 | 53±9 |


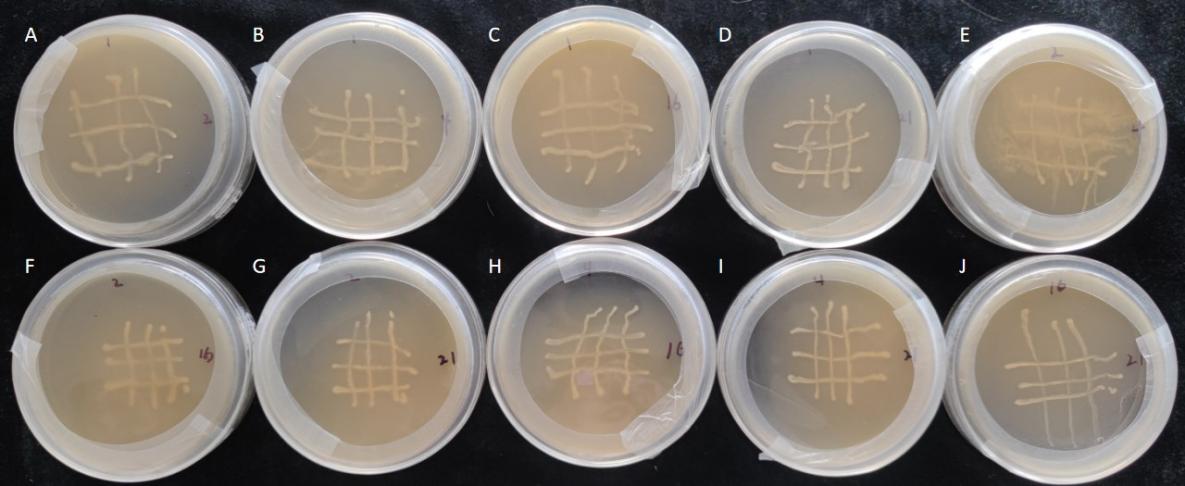


**Suppl. Fig. S1.** Antagonism test among 5 strains of high efficient nitrogen-fixing bacteria *A. mongholicus*

Note: A Petri dish is J1 and J2; B Petri dish is J1 and G4; C Petri dish is J1 and T16; D Petri dish is J1 and T21; E Petri dish is J2 and G4; F Petri dish is J2 and T16; G Petri dish is J2 and T21; H Petri dish is G4 and T16; I Petri dish is G4 and T21; J Petri dish is T16 and T21


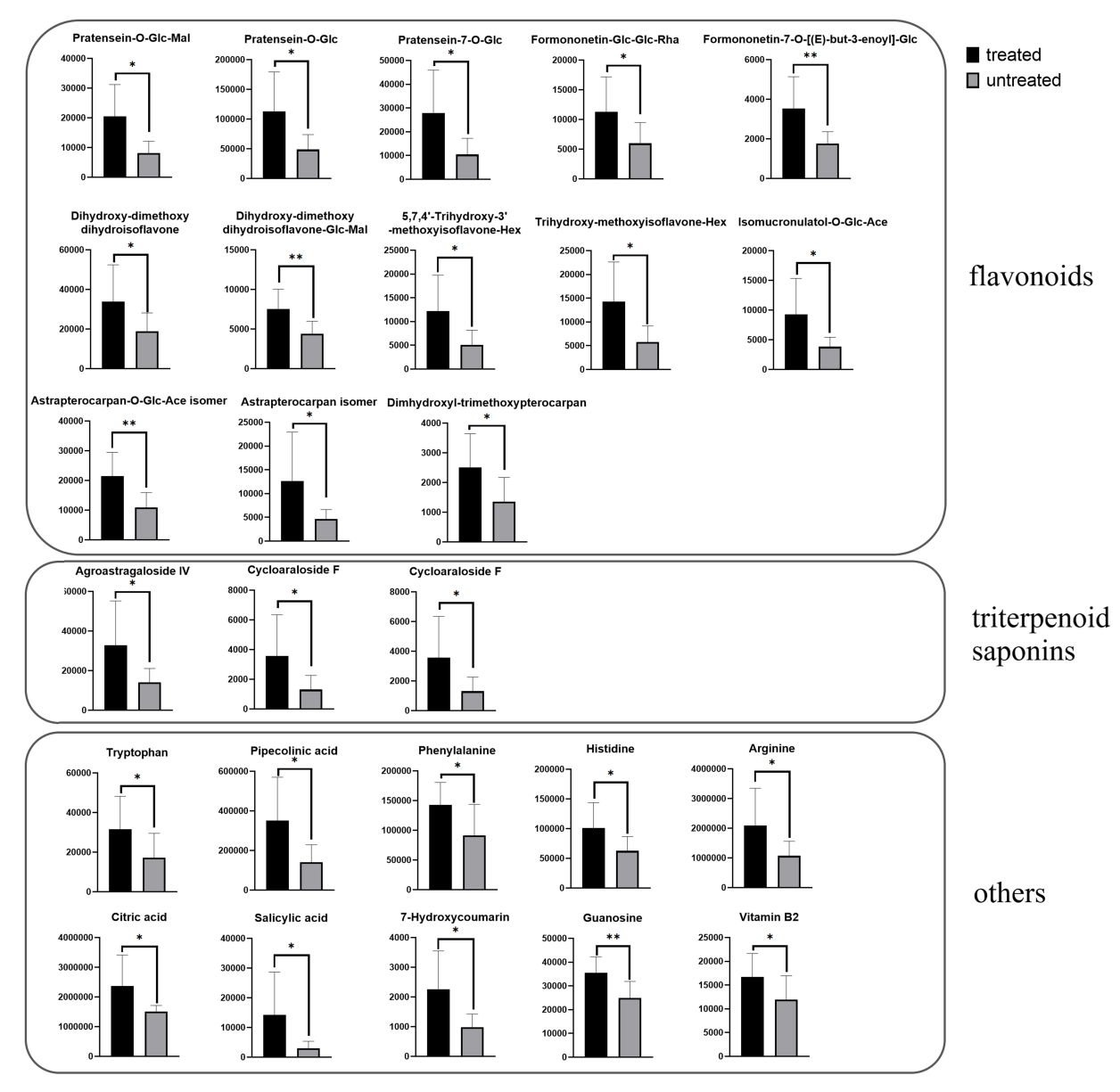


**Suppl. Fig. S2.** Box plots of 26 different compounds.
