# Supplementary figures and images for: Nitrogen-fixing bacteria promote growth and bioactive components accumulation of Astragalus mongholicus by regulating plant metabolism and rhizosphere microbiota
Source: BMC Microbiol. 2024 Jul 15;24:261. doi: 10.1186/s12866-024-03409-y (PMC11247893; doi:10.1186/s12866-024-03409-y)

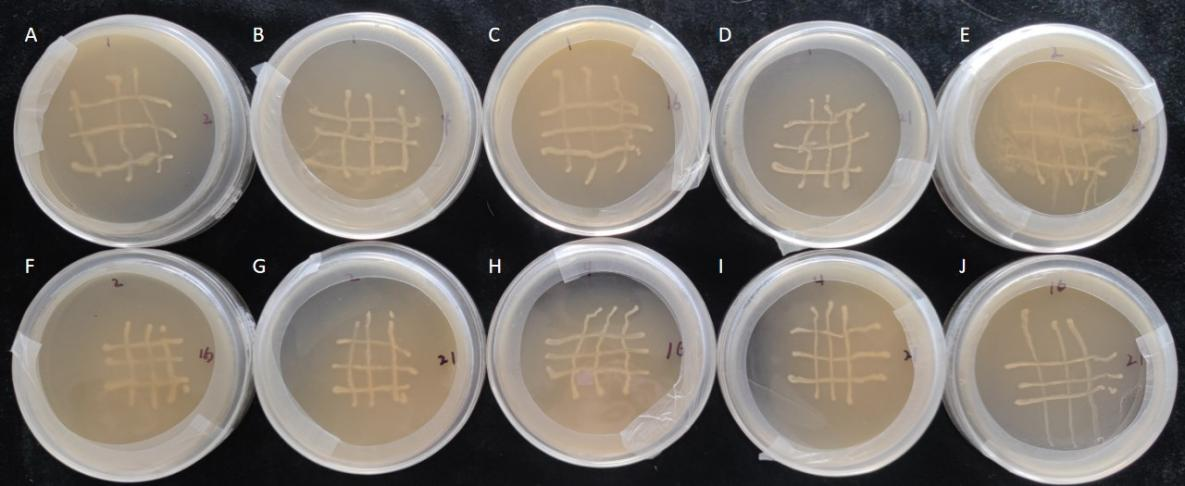

Supplement: Supplementary file 1 — Supplementary Material 1. [file 12866_2024_3409_MOESM1_ESM.zip › Suppl Fig S1.tif]

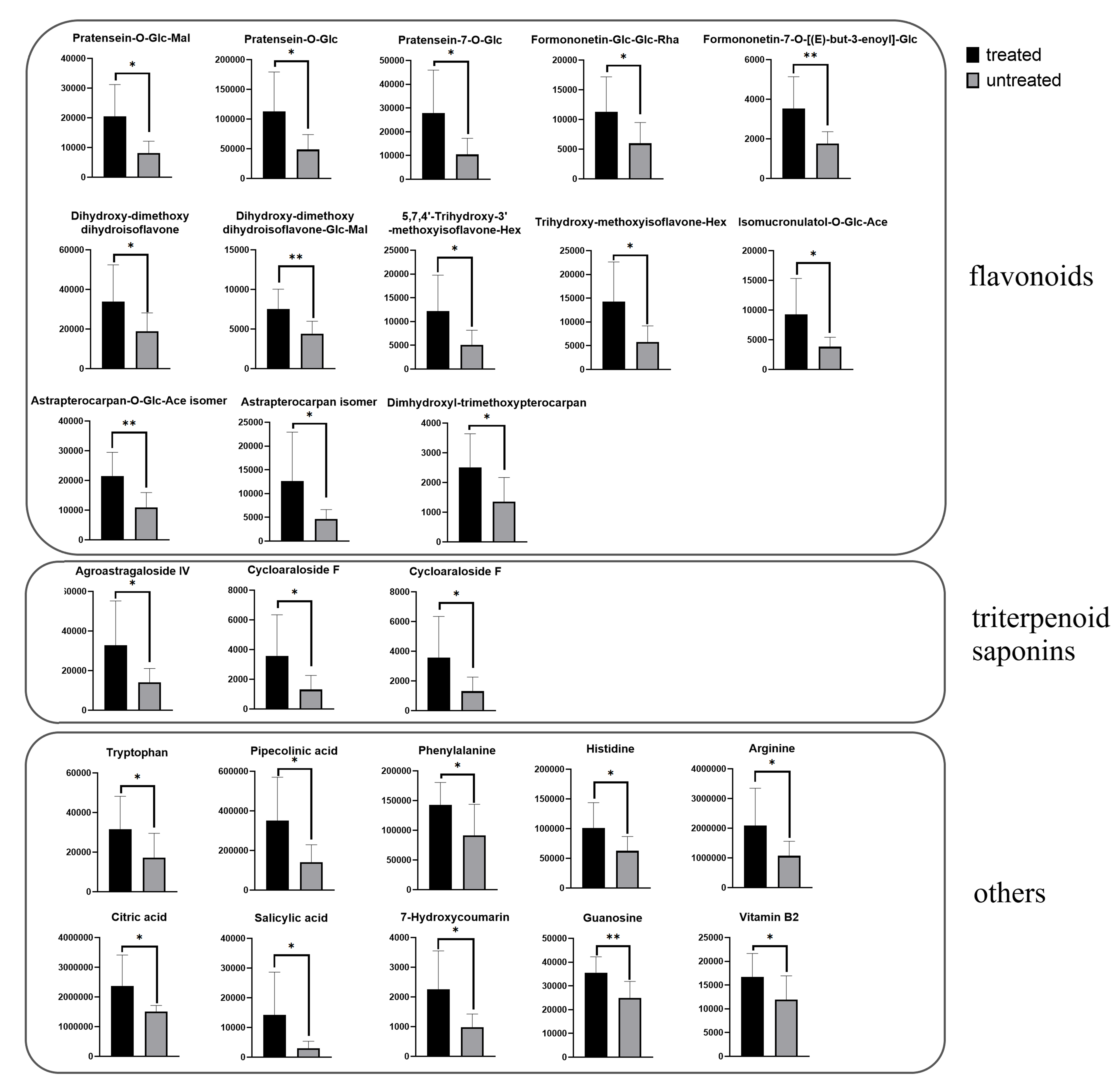

Supplement: Supplementary file 1 — Supplementary Material 1. [file 12866_2024_3409_MOESM1_ESM.zip › Suppl Fig S2.tif]
